# Intercellular wireless communication network between mother and fetus in rat pregnancy-a study on directed and weighted network

**DOI:** 10.1186/s12958-019-0485-8

**Published:** 2019-05-01

**Authors:** Miao Zhao, Tingting Liu, Guangchang Pang

**Affiliations:** 1 Tianjin key laboratory of food biotechnology, Biotechnology & food Science College, Tianjin University of Commerce, Tianjin, 300134 China; 2 I-Mab Biopharma Co.,Ltd, Beijing, 100030 China

**Keywords:** Cytokine, Cell–cell communication network, Communication between mother and fetus, FcγRn

## Abstract

**Background:**

The maternal body forms a wireless communication system with the embryo through the blood circulation system. Obviously, direct sampling from early embryos is damaging. Therefore, we detected changes in the concentrations of 30 signaling molecules in serum from the pregnant rats at the 14 time points, then the intercellular wireless communication network was established, to explore the regularity of signal communication between mother and fetus.

**Method of study:**

We used liquid chip scanning technology to detect 30 signal molecules at 14 time points. Statistical analysis of the data yielded significant change signal molecules. According to the secretory cells and effector cells involved in signal molecules, the communication network of different stages were drawn by using Biograph software.

**Results:**

The process could be divided into 4 periods including early, middle, late pregnancy, and postpartum. In early pregnancy, two immune transformations occur: (a) interleukin-10 (IL-10), interleukin-13 (IL-13) increased at day 5, which promoted immunoglobin G (IgG) secretion, provided protection through the neonatal Fc receptor for IgG (FcγRn) crossing the placental barrier to reach the embryo, achieved T helper 1 (Th1) transformation into T helper 2 (Th2), reduced maternal innate and cellular immunity, and prevented fetal abortion; (b) the fetal heart was fully developed at day 7, with circulatory system established, which provided a platform for intercellular information exchange. The second transformation corresponded to the maternal immune system providing signaling molecules for the embryo to promote Th2 transformation into Th1, thus activating embryonic innate immune cells, and enabling antibody-mediated immune recognition, response and protection. Days 9–19 was a stable period. After 21 days of pregnancy, the maternal body prepared for delivery. The characteristic signaling molecules in the process were monocyte chemotactic protein-1 (MCP-1), IL-10, IL-13, IL-1ɑ, interferon-inducible protein-10 (IP-10), regulated upon activation normal T cell expressed and secreted (RANTES), thyroid stimulating hormone (TSH), IL-2, IL-6, IL-12p70 and IL-18.

**Conclusion:**

Detection of concentration changes of the factors in maternal serum could provide a tool for monitoring, diagnosis, prediction and treatment of embryo differentiation, development and health.

## Background

Embryonic development is a process of strict communication between mother and fetus, and healthy fetal development is significant for procreation and reproduction of a population. Studies have shown that mammalians have regulated embryo-fetal development, that is, embryonic stem cells are strictly induced and modulated by signaling molecules, especially cytokines, during differentiation and whole developmental process [1–3]. In other words, maternal body forms a wireless communication system with the embryo through the blood circulation system, so during embryo development, cytokines provided by the maternal body to the fetus are extremely important for fetal growth and development. Obviously, direct sampling from early embryos is damaging; therefore, in the present study, by detecting changes in the concentrations of 30 signaling molecules in serum from the pregnant rats at the 14 time points, a directed and weighted network, an intercellular wireless communication network between mother and fetus, was drawn by a software developed by our research group. Information exchange between mother and fetus should precede development and differentiation of fetal morphology; therefore, fetal differentiation, development and health could be monitored based on changes in the concentrations of characteristic signaling molecules in maternal serum at each time points, which could help achieve timely prevention and treatment.

## Material and methods

### Animals

All animal procedures in this study were approved by the Biomedical Engineering Institute of Academy of Medical Sciences of China in accordance with the guidelines about Animal Care of the Medical Sciences of China. Female and male Sprague-Dawley rats, aged 60 days (weighing 205-221 g), were purchased from the Animal Center of Academy of Military Medical Sciences of People’s Liberation Army. Rats were maintained under controlled lighting (12 h light-12 h dark cycle) and temperature (22 °C) with ad libitum access to food and water. Female were fed a standard rat chow for 2 weeks before being mated with age-matched Sprague-Dawley males fed the same diet. Copulation was confirmed by the presence of sperm in a vaginal flush; the day of copulation was designated as gestational day 1. After copulation, female and male rats were separated.

### Experimental design and sampling

1 mL blood was collected from the eye sockets of five female rats before being mated as the control. Blood sample was collected respectively from the five female rats at 3 pm on the gestation days 1, 3, 5, 7, 9, 11, 13, 15, 17, 19, 21, 23, and days 1 and 3 after delivery; a total of 14 time points (70 female rats were used). After clottting, the samples were centrifuged at 1000 r/min for 10 min and the serum was separated. Serum samples were stored at − 20 °C for later use.

### Detection of main signaling molecules

The Liquichip workstation liquid protein chip system, a new type of protein research and analysis platform, can be used to simultaneously detect multiple molecules in same sample, as the flexible Muti-Analyte Profiling (xMAP) technology. The Liquichip workstation organically integrates colored microspheres, laser technology, fluidics and the latest mathematical signal processors, and computer programming algorithms. This technology has the characteristics of unparalleled detection specificity, sensitivity and operability.

We select the appropriate number of antibodies based on common cytokines related to embryonic growth and differentiation. However, some cytokines have a response concentration below the detection limit and are therefore not selected. Therefore in this experiment, a Millipore rat cytokine/chemokine kit was used to detect 30 signaling molecules: Eotaxin, granulocyte macrophage-colony-stimulating factor (GM-CSF), granulocyte colony-stimulating factor (G-CSF), IL-1α, MCP-1, Leptin, macrophage inflammatory protein 1 alpha(MIP-1α), IL-4, IL-1β, IL-2, IL-6, IL-13, IL-10, IL-12p70, IL-5, interferon gamma (IFN-γ), IL-17, IL-18, IP-10, growth-related oncogene (GRO), RANTES, tumor necrosis factor-alpha(TNF-α), vascular endothelial growth-factor(VEGF), adrenocor ticotropic hormone(ACTH), brain derived neurotrophic factor(BDNF), follicle-stimulating hormone(FSH), growth hormone(GH), luteinizing hormone(LH), prolactin(PRL) and TSH (As shown in Table 1).

**Table 1:** Abbreviations and full-names correspondence table

Abbreviations	Names
IL	interleukin
IgG	immunoglobin G
FcγRn	the neonatal Fc receptor for IgG
Th1	T helper 1
Th2	T helper 2
MCP-1	monocyte chemotactic protein-1
IP-10	interferon-inducible protein-10
RANTES	regulated upon activation normal T cell expressed and secreted
TSH	thyroid stimulating hormone
GM-CSF	granulocyte macrophage-colony-stimulating factor
G-CSF	granulocyte colony-stimulating factor
MIP-1α	macrophage inflammatory protein 1 alpha
IFN-γ	interferon gamma
GRO	growth-related oncogene
TNF-α	tumor necrosis factor-alpha
VEGF	vascular endothelial growth-factor
ACTH	adrenocorticotropic hormone
BDNF	brain derived neurotrophic factor
FSH	follicle-stimulating hormone
GH	growth hormone
LH	luteinizing hormone
PRL	prolactin
TSH	thyroid-stimulating hormone

### Statistical analysis

The SPSS 17.0 was used to pass the normal distribution test (Kolmogorov-Smirnov), and the data conforming to the normal distribution was compared with the control experiment results by the comparative t-test. The non-conformity data was subjected to the nonparametric test, and the 95% confidence interval was selected (As shown in Table 3). All data were expressed as the mean ± standard deviation.

### Establishment of an intercellular communication network model

Based on the changes in the signal molecules in the serum, the rates of change in the content of the signal molecule *i* (one of 30 signal molecules) can be expressed using the following equation:

1 $$ {\xi}_i/\%=\frac{1}{5}\sum \limits_{j=1}^{n=5}\frac{c_{ij}-{c}_{ij0}}{c_{ij0}}\times 100 $$

c*ij* represents the concentration of signal molecule *i* after pregnancy and c*ij*0 represents the concentration of signal molecule *i* in the control test.

Here, it was to map out the virtual cytokine network in vivo according to the rates of change in the content of 30 signal molecules to describe the effect of nonpregnant group(compared to pregnant group). BiologyGraph mapping software developed by our research group was used to draw the intercellular signal molecule network diagram among the secretion cells and target cells that was available from the cytokine database (The Cytokines Online Pathfinder Encyclopedia COPE, http://www.copewithcytokines.de/. This database is only used to query the secretion cells and effector cells of a certain cell, and is used to construct a intercellular wireless communication.). The communication effect between all the secretion cells and target cells could be expressed using the following formula:

2 $$ {E}_{st}/\%=\sum \limits_{i=1}^{m=30}{s}_i{t}_i{p}_i{\xi}_i $$

*Est* represents the communication effect between secretion cells and target cells; *s* represents whether the cell was able to secrete cytokines *i*, the value should be 1, or else 0; *t* represents whether cell was the target cell of cytokines *i*, the value should be 1, − 1, or else 0; *p* represents the statistical significance of cytokine (*P* < 0.05), the value should be 1, or else 0.

The intercellular cytokine network diagram was drawn according to the *Est* values (Table 2). The thickness of lines represents the strength of the transmission signals (*Est* values), and the color of the lines had different meanings: the red line represented intensity of the communication and the blue line for abating the communication. This network was a directed-weighted network, in which the cells were nodes, the signal transmissions were lines [4–6]. The whole strength of the network was the sum of the strength of lines, which was an important index to reflect the network intercellular communication ability. The whole strength of the network was categorized into positive network strength *S + network*, negative network strength *S-network*, and total network strength *Snetwork* [7], due to the positive and negative line strengths.

**Table 2:** Communication effect of the secretion cells on the target cells

Cells		Target cells of cytokines			
		Target cell1	Target cell2	…	Target celln
Secretion cells of cytokines	Secretion cell1	E_11_	E_12_	…	E_1n_
	Secretion cell2	E_21_	E_22_	…	E_2n_
	…	…	…	…	…
	Secretion celln	En_1_	En_2_	…	E_nn_

*n* represents the total number of cells

E_*nn*_ represents the communication effect of the secretion cells on the target cells

## Results

### Signaling molecules with significant changes

Compared to the serum levels of the signal molecules before pregnancy, at least one of the 30 signal molecules showed significant changes at 12 of the 14 tested time points, with the 3rd and 11th day after pregnancy as two exceptions. During this period, 20 signal molecules showed significant changes (Table 3).

**Table 3:** Signal molecules that showed significant changes during this period

Gestation period	Cytokines	*P*-value	Rate of change in concentration	Statistical analysis method
E1	LH	0.021*	0.694	Comparative t-test
	BDNF	0.013*	−0.425	Comparative t-test
E5	MCP-1	0.012*	23.5	Nonparametric test
	IL-10	0.016*	0.918	Comparative t-test
	IL-13	0.012*	119.0	Comparative t-test
	GRO	0.063	8.47	Comparative t-test
E7	Leptin	0.029*	1.46	Comparative t-test
	IL-1α	0.037*	14.4	Comparative t-test
	IP-10	0.041*	5.11	Comparative t-test
	IFN-γ	0.059	26.4	Comparative t-test
E9	GH	0.045*	4.05	Comparative t-test
E13	Leptin	0.093	1.12	Comparative t-test
E15	LH	0.047*	−0.590	Comparative t-test
E17	RANTES	0.002**	−0.279	Comparative t-test
	GH	0.039*	4.19	Comparative t-test
	LH	0.080	−0.519	Comparative t-test
E19	Leptin	0.005**	1.91	Comparative t-test
	TSH	0.001**	1.79	Comparative t-test
	ACTH	0.049*	−0.477	Comparative t-test
	GH	0.065	3.72	Comparative t-test
E21	ACTH	0.002**	−0.757	Comparative t-test
	BDNF	0.005**	−0.484	Comparative t-test
	IL-2	0.017*	2.77	Comparative t-test
	IL-6	0.013*	0.755	Comparative t-test
	IL-12P70	0.017*	1.11	Comparative t-test
	IL-18	0.018*	3.33	Comparative t-test
	IFN-γ	0.044*	29.9	Comparative t-test
	Leptin	0.054	1.28	Comparative t-test
	GRO	0.060	8.55	Comparative t-test
E23	ACTH	0.036*	−0.507	Comparative t-test
	BDNF	0.008**	−0.458	Comparative t-test
D1	VEGF	0.008**	1.61	Nonparametric test
	BDNF	0.002**	−0.531	Comparative t-test
	ACTH	0.022*	−0.559	Comparative t-test
D3	PRL	< 0.001**	4.70	Comparative t-test
	ACTH	0.023*	−0.552	Comparative t-test
	LH	0.030*	−0.648	Comparative t-test

*P < 0.05, **P < 0.01

E (during the period of pregnancy), D (post partum)

**Fig. 1:**
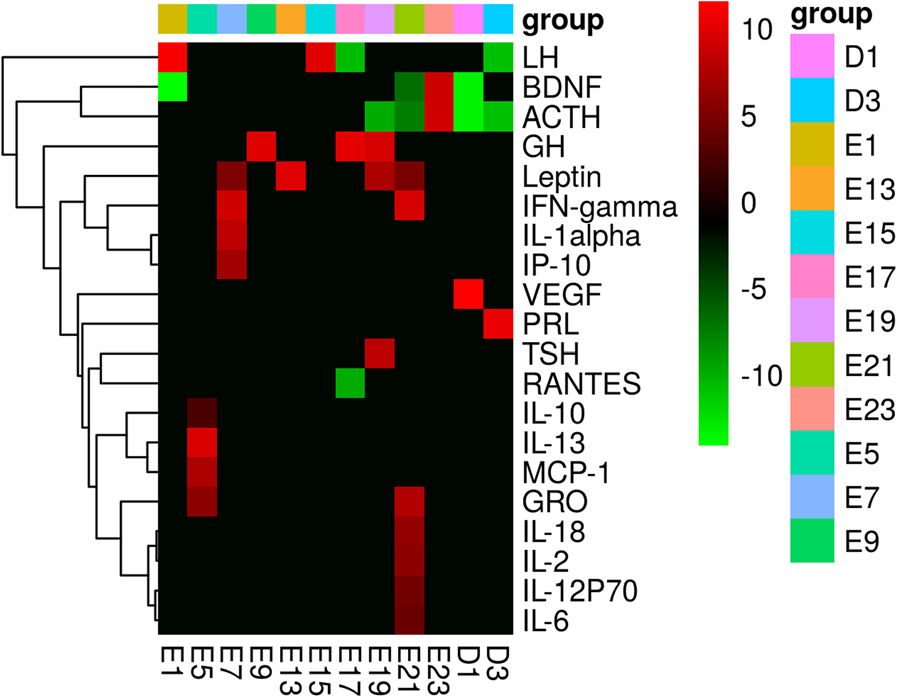
Signaling molecules are altered at different time points to adapt to the needs of physiological activities. The signal molecules showed the most changes in the third trimester and moderate changes in the first trimester, but were relatively stable during the second trimester

As shown in Fig. 1, as gestation proceeds, levels of the signal molecules change to satisfy the physiological requirements at different stages. The signal molecules showed the most changes in the third trimester and moderate changes in the first trimester, but were relatively stable during the second trimester.

### Changes in the intercellular wireless communication network

In order to systematically analyze signal communication networks between the mother and the fetus, a wireless communication network between the mother and the fetus was built (Figs. 3, 4, 5 and 6).

### Early pregnancy (days 1–7; equivalent to the first trimester of human pregnancy)

As shown in Table 4 [8], at pregnancy day 1 (equivalent to the 1st month of human pregnancy), the signaling molecules LH and BDNF were significantly increased and decreased, respectively, compared with the pre-pregnancy levels. As shown in Fig. 3a, there were 12 cell types involved in intercellular wireless communication network. During this period, sperm-egg binding and fusion formed the fertilized egg, which moved from the fallopian tube to the uterus, and constantly sent stimulation signals to the endometrium. This promotes an acceptance state for the endometrium, leading to a smooth progression of blastocyst implantation. LH had the effects of promoting oocyte maturation and ovulation. Kawamura K showed that low BDNF concentration in maternal serum during early pregnancy plays an important role in follicle maturation and oocyte formation in the maternal body [9]. In addition, BDNF in the circulatory system might be reduced by LH inhibition.

**Table 4:** Time correspondence between rats and humans in the whole process

	The pregnancy stages										Post partum	
Rat	The 1st day	The 5th day	The 7th day	The 9th day	The13^th^ day	The15^th^ day	The17^th^ day	The19^th^ day	The21st day	The23^rd^ day	The 1st day	Th3 ^rd^ day
Human	The 1^st^ month	The 2nd month	The 3rd month	The 4th month	The 5th month	The 6th month	The 7th month	The 8th month	The 9th month	The10^th^ month	The 1st month	

**Fig. 2:**
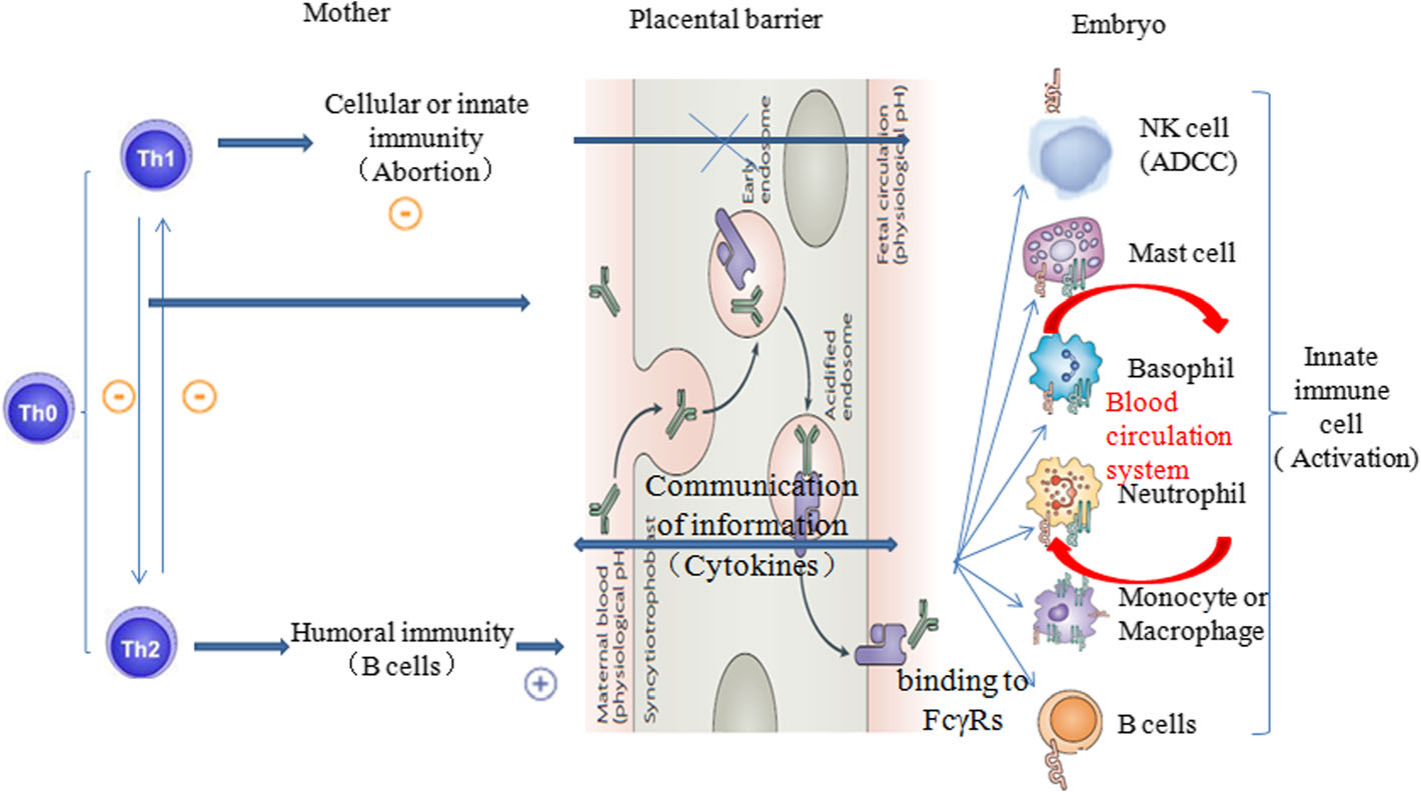
At pregnancy day 5 in rats, maternal innate and cellular immune cells cannot cross the placental barrier into the fetus to provide protection. Therefore, in order to prevent fetal abortion, the cellular immune activity mediated by maternal Th1 is inhibited, thus enhancing humoral immunity. The aim is to activate B cells to produce large amounts of IgG, which cross the placental barrier into the fetus through FcγRn and provide protection, i.e. Th1 transformation into Th2 occurs. At pregnancy day 7, the fetal heart is fully developed, and the embryo has its own circulatory system; at this time, FcγRn bound maternal IgG in the fetus exchange with FcγRs on the surface of innate immune cells in the fetus. In order to activate fetal innate immune cells, transformation of Th2 into Th1 is completed in the maternal immune system, so that the embryo uses maternal IgG to mediate its innate immune protection and to provide protection for itself. (Some of the pictures in this figure are from Roopenian DC [8])

**Fig. 3:**
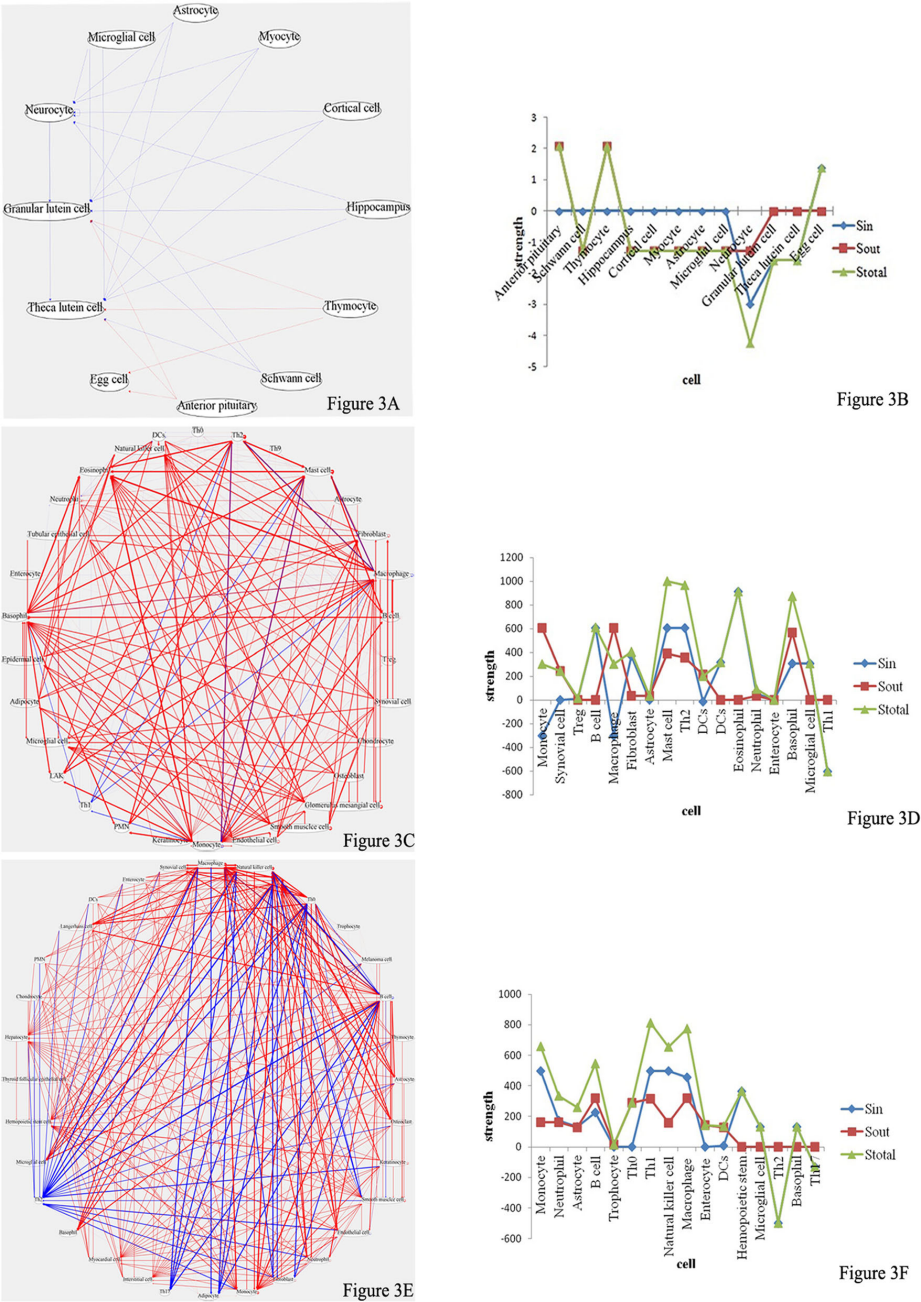
**a**, **c** and **e**, Intercellular wireless communication networks of pregnancy days 1, 5, and 7, respectively. **b**, **d** and **f**, Cellular strengths at pregnancy days 1, 5, and 7, respectively

Compared with pre-pregnancy levels, the signaling molecules MCP-1, IL-10, IL-13 and GRO showed increased amounts at pregnancy day 5 (equivalent to the 2nd month of human pregnancy) (Table 4). As shown in Fig. 3c, a total of 30 cell types were involved in intercellular communication network. The roles of cytokines were quite complex, but according to the intercellular communication network map, their overall effect was maternal Th1 inhibition (S_*in*_ = − 606.934) and Th2 activation (S_*in*_ = 606.934). As shown in Fig. 2, during this period, the fetus had no fetal heart and did not have the ability to protect itself. It was a heterogenous tissue in the maternal body. Meanwhile, maternal immune cells cannot cross the placental barrier to provide fetal protection. Therefore, if maternal innate and cellular immune activities are too high, fetal abortion would occur. During this period, when maternal innate and cellular immune activities are inhibited, humoral immune responses are enhanced significantly, thus activating B cells to produce IgG molecules, which cross the placental barrier and are transported to the fetus through FcγRn, providing fetal protection [10]. This study showed a rate of concentration change for IL-13 of 119 (*P* < 0.05). A study showed that IL-13 is a Th2-type cytokine, which can inhibit Th1 cells, reduce the maternal immune rejection of the fetus, activate B cells to produce large amounts of IgG and complete Th1 transformation into Th2 [11]; therefore, IL-13 plays an important role in preventing fetal abortion. In addition, the rate of change in IL-10 concentration was 0.918 (P < 0.05). A study demonstrated that IL-10 inhibits natural killer (NK) cells at the maternal fetal interface as well as Th1-type cytokines such as IFN-γ and IL-2, thus indirectly inhibiting the maternal immune rejection of the fetus [12].

As shown in Table 3, at pregnancy day 7 (equivalent to the 3rd month of human pregnancy), compared with pre-pregnancy levels, signaling molecules with significant increase included Leptin, IL-1α, IP-10 and IFN-γ. The rates of concentration change for IL-1α, IP-10 and IFN-γ were 14.4 (*P* < 0.05), 5.11 (P < 0.05) and 26.4 (*P* < 0.1), respectively; leptin, IP-10 and IFN-γ are all Th1-type cytokines, which increase cellular immune activities in the maternal immune system. In addition, the rate of concentration change for leptin was 1.46 (P < 0.05); leptin promotes the catabolism of adipocytes in the maternal body and reserves nutrition for embryonic development. As shown in Fig. 3e, a total of 33 cell types were involved in intercellular communication network. Th1 activity obviously relieved the inhibitory state in comparison with that obtained at pregnancy day 5 (S_*in*_ = 496.98); meanwhile, the corresponding Th2 activity was decreased significantly (S_*in*_ = − 498.44). As shown in Fig. 2, the fetal heart has been fully developed, with the fetus having circulatory system, which provided an autonomic communication platform for energy, material and information among body tissues and cells. At this time, IgG antibodies provided by the maternal immune system can continue to bind FcγR on the surface of innate immune cells (e.g. neutrophils, mononuclear macrophages and NK cells) in the embryo through the circulatory system, and mediate the immune protective effect of the fetus [13]. During this period, cytokines secreted by the maternal immune system for innate and cellular immunity activation can cross the placenta into the fetus, and promote innate and cellular immune development, differentiation and activity of the fetus through the circulatory system. Therefore, the second Th1 transformation into Th2 in the maternal immune system occurred, aiming to activate innate immune activities in the embryo. When fetal innate immune cells are activated, the embryo can mediate its innate immune protection by maternal IgG, for example, NK cell-mediated antibody-dependent cell-mediated cytotoxicity(ADCC).

### Mid-pregnancy (days 9–19, equivalent to pregnancy months 4–8 in humans)

**Fig. 4:**
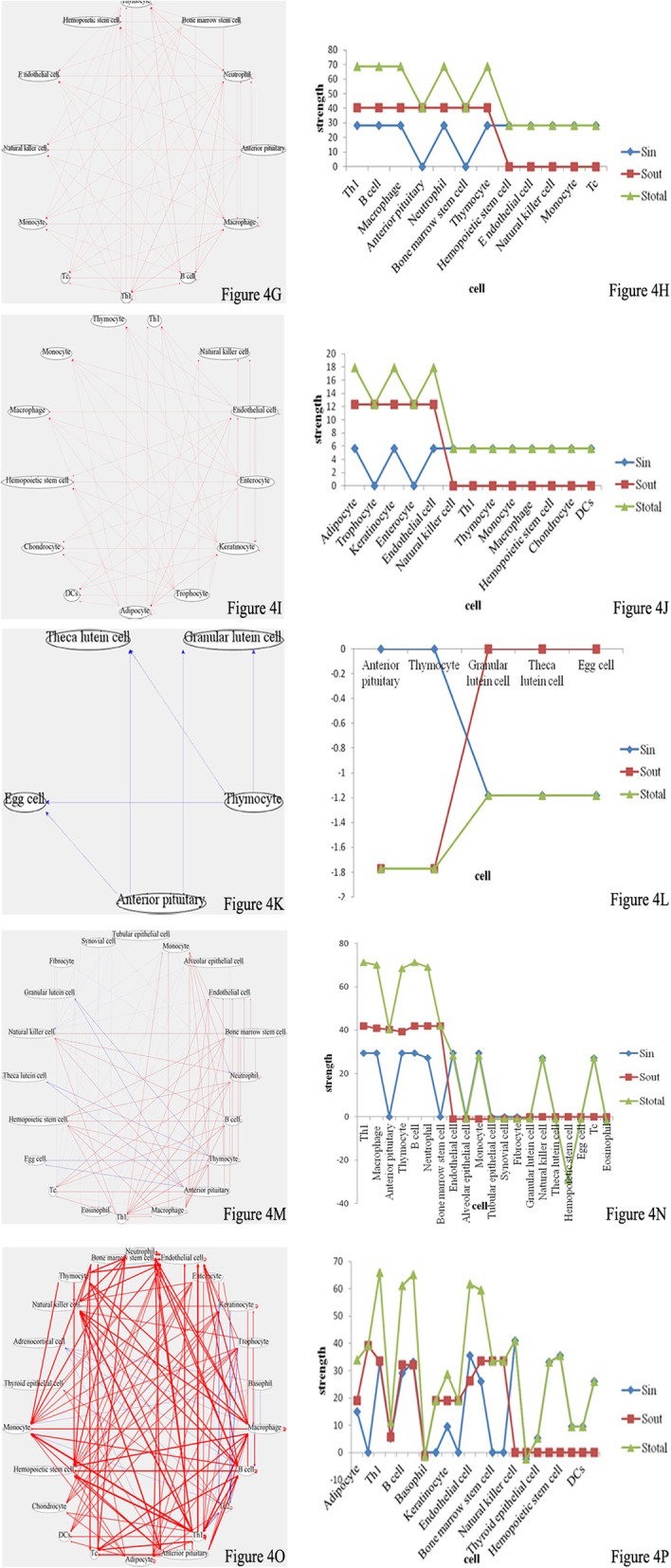
**g**, **i**, **k**, **m** and **o**, Intercellular wireless communication networks of pregnancy days 9, 13, 15, 17 and 19, respectively. **h**, **j**, **l**, **n** and **p**, Cellular strengths at pregnancy days 9, 13, 15, 17 and 19, respectively

As shown in Table 4, during the period between pregnancy days 9 and 19 in rats (equivalent to pregnancy months 4–8 in humans), mother and fetus were basically in a stable state (Fig. 4). GH and Leptin secretion levels were increased, promoting muscle growth and adipocyte metabolism, which resulted in enhanced cell division and growth. The two cytokines provided by the mother to the fetus were beneficial to fetal growth and development. In addition, Th2 activity was basically stable during this period, and Th1 activity was significantly decreased in comparison with that of pregnancy day 7, but still slightly higher than normal levels. Because fetal innate and cellular immunity had gradually matured at this time, the mother did not need to provide the fetus with the required cytokines to enhance cellular immune activity; meanwhile, Th1 activity was properly adjusted to reduce maternal immune rejection of the fetus and avoid fetal abortion.

### Late pregnancy (days 21–23, equivalent to pregnancy months 9–10 in humans)

**Fig. 5:**
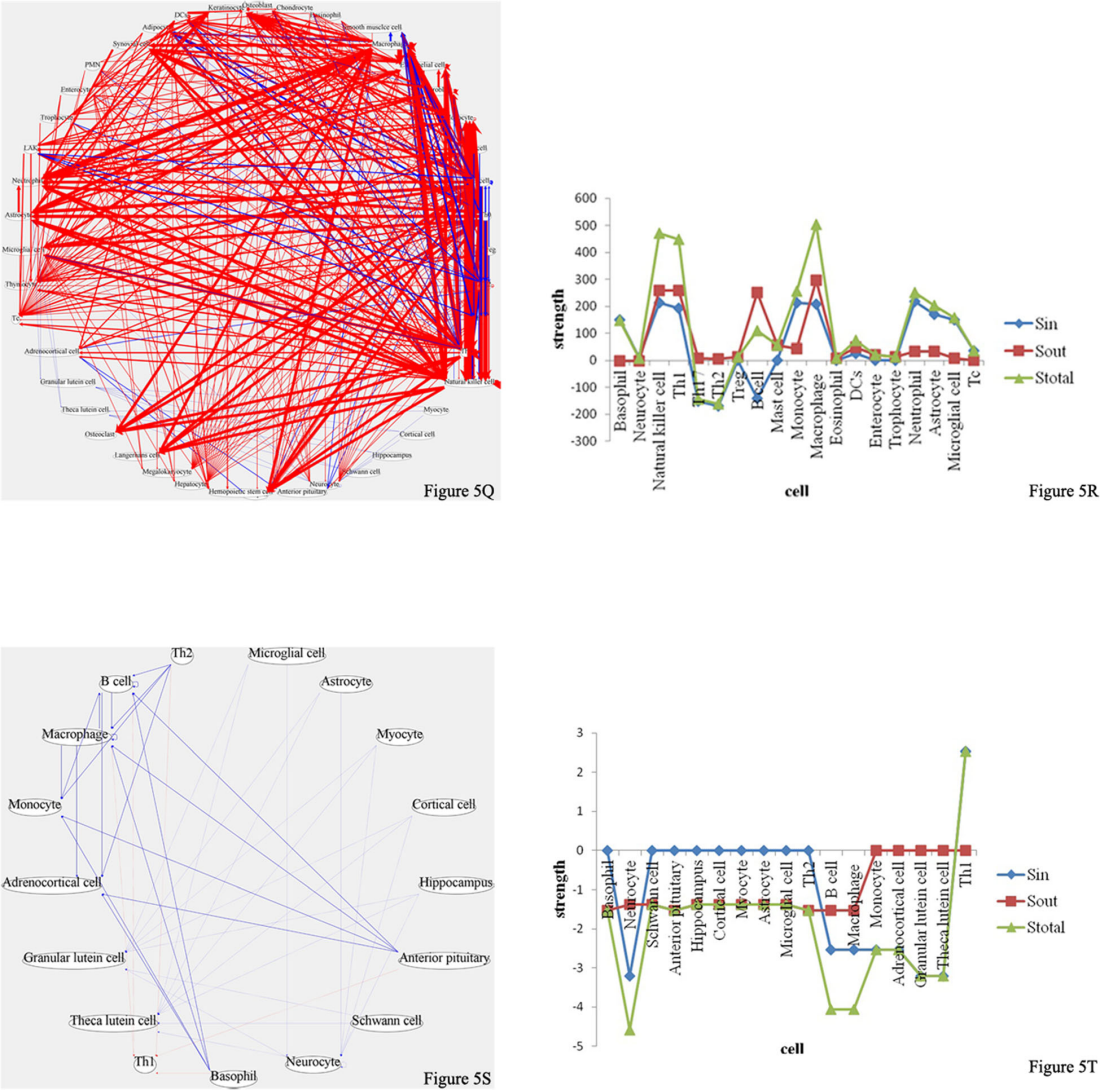
**q** and **s**, Intercellular wireless communication networks of pregnancy days 21 and 23, respectively. **r** and **t**, Cellular strengths at pregnancy days 21 and 23, respectively

As shown in Table 4, at pregnancy day 21 (equivalent to pregnancy month 9 in humans), IL-2, IL-6, IL-12P70, IL-18, IFN-γ, Leptin and GRO levels were significantly increased, while ACTH and BDNF amounts were markedly reduced, compared with pre-pregnancy levels. At this time, maternal Th1 activity was rapidly activated, which promoted immune protection of mother and fetus during childbirth, preparing for delivery (Fig. 5). In addition, studies have shown that IL-2, IL-6 and IL-18 are all related to uterus expansion close to delivery [14].

At pregnancy day 23, ACTH and BDNF levels were markedly reduced. As shown in Fig. 5, the activity of maternal nerve cells was inhibited, indicating that the mother was about to give birth.

### Postpartum

**Fig. 6:**
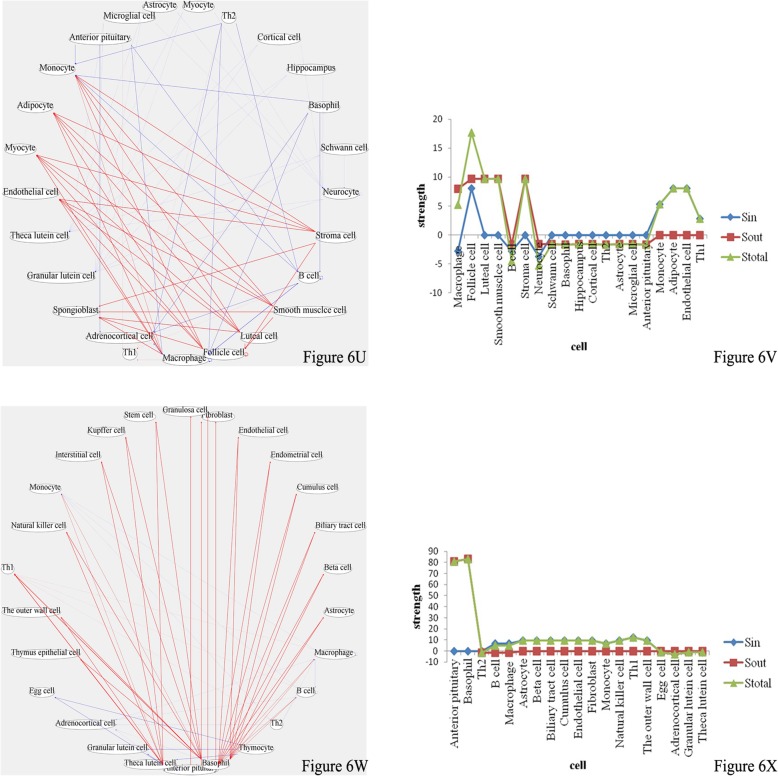
**u** and **w** are intercellular wireless communication networks of days 1 and 3 postpartum, respectively. **v** and **x** are cellular strengths at days 1 and 3 postpartum, respectively

At 1 day postpartum, due uterine tissue damage caused by delivery, massive hemorrhage in the maternal body occurred. As shown in Fig. 6, VEGF levels were increased significantly, activating endothelial cells, smooth muscle cells and so on, and repairing the wounded tissue. Meanwhile, nerve cells continued to be inhibited.

At 3 days postpartum, the maternal body entered lactation period, and PRL increased significantly, promoting and maintaining maternal lactation.

### Characteristic signaling molecules at each time points of pregnancy

**Fig. 7:**
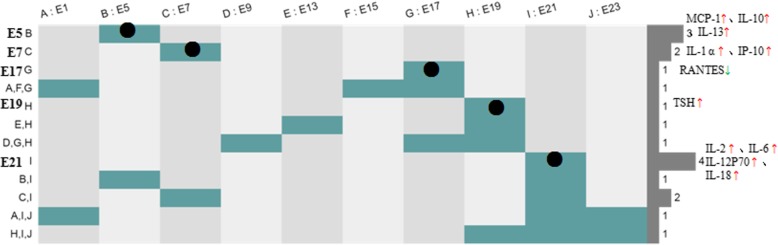
Characteristic signaling molecules with significant changes at pregnancy days 5, 7, 17, 19, and 21. Three characteristic signaling molecules, including MCP-1, IL-10 and IL-13, are increased significantly at pregnancy day 5. At day 7, IL-1α and IP-10 are markedly increased. At day 17, RANTES is the characteristic signaling molecule showing decreased levels. At day 19, TSH is the characteristic molecule with increased amounts. At day 21, 4 characteristic signaling molecules, including IL-2, IL-6, IL-12p70 and IL-18, are increased significantly

As shown in Fig. 7, the characteristic signaling molecules altered at pregnancy day 5 were MCP-1, IL-10 and IL-13, with rates of concentration change of 23.5 (*P* < 0.05), 0.918 (P < 0.05) and 119 (P < 0.05), respectively. At pregnancy day 5, in order to reduce immune rejection of embryo and prevent embryo abortion, Th1 transformation into Th2 in the maternal immune system occurred; therefore, humoral immunity was enhanced. A study demonstrated that IL-13 plays an important role in inhibiting Th1 activity and activating B cells to produce IgG, could enter embryo through placental barrier to provide fetal protection. Surveys have shown that decreased IL-13 concentration in maternal body in early pregnancy could lead to embryo abortion [15]. Moreover, other studies have suggested that macrophages in decidual tissues in early pregnancy secrete large amounts of IL-10; compared with those of normal pregnant women, IL-10 levels in decidual tissues of women with spontaneous abortion are decreased [16, 17]. A study also suggested that IL-10 participates in production of flavonoids and progesterone, which play important roles in maintaining normal pregnancy. MCP-1 is a chemokine [18]. A study demonstrated that embryo implantation includes blastocyst localization, adhesion and invasion in the endometrium [19]. During blastocyst localization, blastocyst-endometrium communication depends on soluble mediators, such as MCP-1, which conduct a two-way communication between blastocyst and endometrium; therefore, chemokines are first produced by endometrium and reach a certain peak during embryo implantation [14]. MCP-1 concentration decrease in early pregnancy might cause embryo implantation errors; therefore, this chemokine can help assess whether or not embryo is normally implanted in the endometrium. Overall, We can monitor and diagnose the embryo by measuring these three signaling molecules concentrations, and even provide treatment, indicating the clinical significance of these findings.

At pregnancy day 7, characteristic signaling molecules included IL-1α and IP-10, with rates of concentrations change of 14.4 (*P* < 0.05) and 5.11 (P < 0.05), respectively. At pregnancy day 7, the fetal heart was fully developed and fetus had circulatory system, which provided a platform for intercellular information exchange. At this time, innate and cellular immune activities in embryo needed to be activated, and maternal IgG molecules were used to mediate the immune response to foreign antigens, thus providing fetal protection. Therefore, maternal body needed to produce Th1-type cytokines, such as IL-1α and IP-10, and to complete Th2 transformation into Th1. In addition, a study demonstrated a mutual promotion effect between IP-10 and IFN-γ to some extent, which can decrease Th2 activity [20]. When these two cytokines are decreased in levels, fetal innate immunity is likely not activated, and the fetus is unable to provide protection for itself, resulting in abortion. During pregnancy, fetal health and development can be monitored and even treated by detecting the concentrations of these two cytokines.

At pregnancy day 19, characteristic signaling molecule with significant increase was TSH; the rate of concentration change was 1.79 (*P* < 0.01). A study pointed out that TSH content in maternal body in early pregnancy is the lowest, increasing gradually after entering stable period [21]. This might be due to the amounts of thyroid hormone from maternal body increasing as the fetus develops gradually in mid-pregnancy; the negative feedback regulation significantly increases TSH levels in maternal body [22]. By measuring TSH levels in maternal body, we could assess fetal growth and development.

At pregnancy day 21, characteristic signaling molecules with significant increase were IL-2, IL-6, IL-18 and IL-12p70, with rates concentration change of 2.77 (*P* < 0.05), 0.755 (P < 0.05), 3.33 (P < 0.05), and 1.11 (P < 0.05), respectively. IL-2, IL-6 and IL-18 belong to Th1-type inflammatory cytokine family. Sennstrom suggested that when maternal body is close to delivery, IL-6 amounts are increased significantly, probably because mature expansion of the cervix is a physiological inflammatory reaction [23]. During whole pregnancy, IL-18 amounts were higher, and after childbirth started, it further increased until delivery end, before decreasing thereafter.

### Correlation analysis of the whole process at different time points

**Fig. 8:**
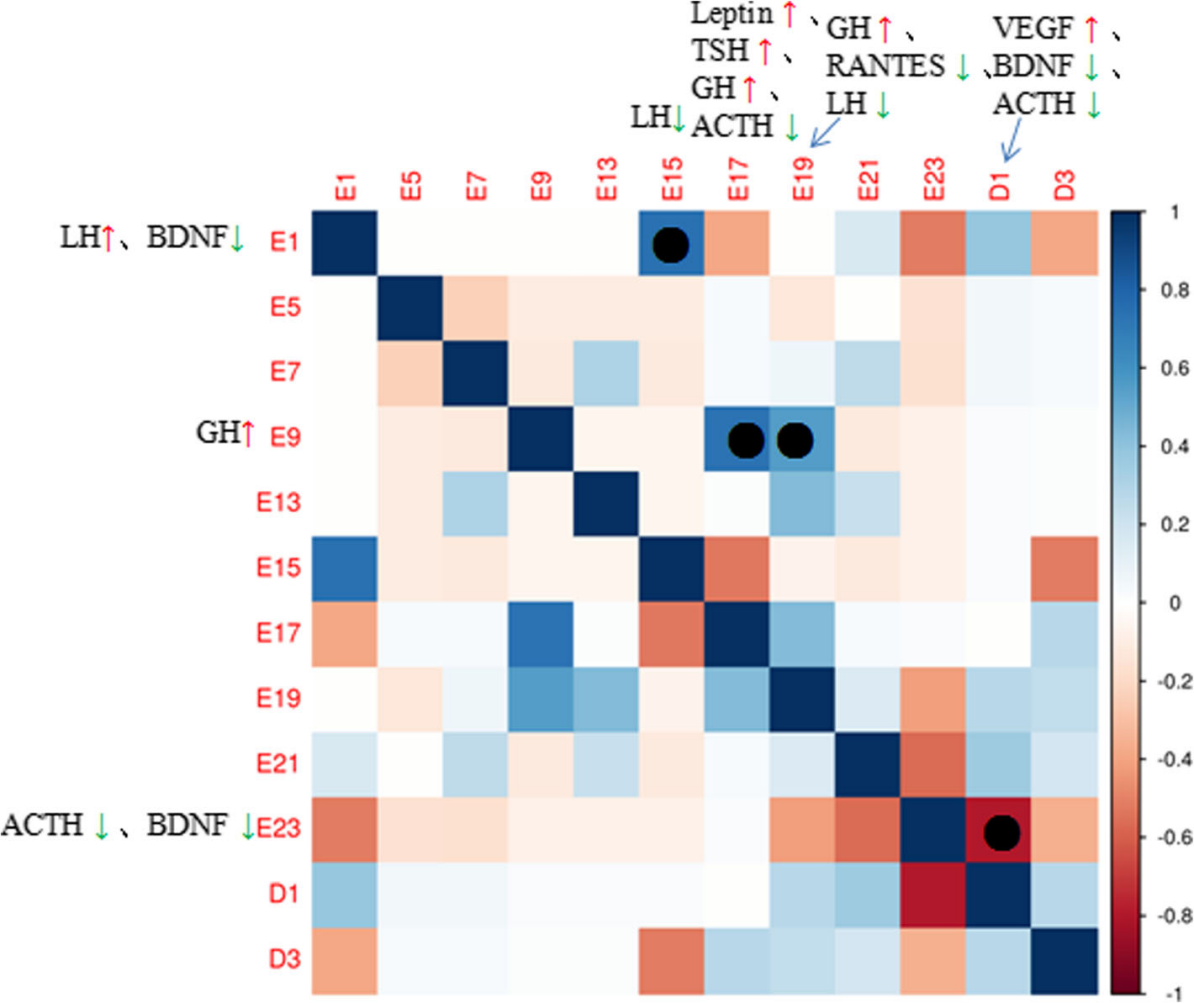
During pregnancy and postpartum, day 1 of pregnancy is positively correlated with day 15 of pregnancy; day 9 is positively correlated with days 17 and 19 of pregnancy, respectively; day 23 of pregnancy (antenatal) is negatively correlated with postpartum

As shown in Fig. 8, there was a significant negative correlation between day 23 of rat pregnancy and day 1 postpartum, because VEGF was significantly increased at 1 day postpartum, with a rate of concentration change of 1.61 (*P* < 0.01). VEGF is one of the most important angiogenic factors, and also a major cytokine regulating permeability and growth of endometrial blood vessels. In the present study, at 1 day postpartum, VEGF levels in maternal body were increased significantly. It might be that the maternal endometrium was traumatized postpartum, with large amounts of VEGF needed for repair.

A significant positive correlation was found at pregnancy days 9, 17 and 19. During this period, GH was altered significantly, with rates of concentration change of 4.05 (*P* < 0.05), 4.19 (*P* < 0.05) and 3.72 (*P* < 0.1), respectively. As mentioned above, during this period (pregnancy days 9–19) embryo grows rapidly, and maternal body must secrete large amounts of GH to promote fetal growth and development. Therefore, fetal growth and development could be monitored by detecting GH in maternal serum. In case of low GH content, fetal growth could be promoted by injection of adequate GH amounts.

There was a highly positive correlation between pregnancy days 1 and 15, and LH changed significantly on both days, i.e. 0.694 (*P* < 0.05) and − 0.59 (*P* < 0.05), respectively. In early pregnancy, LH was increased, playing an important role in oocyte maturation and ovulation; at day 15, LH was decreased significantly.

## Discussion

This study showed that communication of signaling molecules (such as cytokines, chemokines, hormones) between mother and fetus mediated the strict interactions of the fetus at various time points of implantation, differentiation, development and delivery, to maintain balance and adaptation of immune and endocrine activities between mother and fetus. In early pregnancy, IL-6 levels were decreased (not significantly), but increased significantly at day 21 of pregnancy. The possible mechanism is that in early pregnancy, IL-6 decreases in order to prevent maternal immune rejection of embryo; however, with pregnancy time, IL-6 amounts in maternal serum increases gradually. In late pregnancy, cervical collagen fibers were swollen, curled and fractured, while the cervix was softened, shortened and relaxed, similar to acute inflammation. These results corroborated Barrat [24]. Sennstrom et al., who found a gradual IL-6 level increase in amniotic fluid as the number of pregnancy days increases [23].

TNF-α regulates corpus luteum throughout the whole pregnancy, and appropriate amounts of TNF-α in pregnant women can promote catabolism. TNF-α in fetus plays an important role in the proliferation, division and immune protection of embryonic cells [25, 26]. As shown above, with the progression of pregnancy, TNF-α levels in rat serum were gradually decreased, but not significantly in comparison with pre-pregnancy amounts. Monzón-Bordonaba F et al. confirmed that low TNF-α concentration can promote energy metabolism and embryonic development in pregnant women, and improve the synthesis of progesterone and chorionic gonadotropin, which is beneficial to reduction of the extracellular matrix of decidual cells and placental implantation [27]. This plays an important role in the maintenance of pregnancy. Large amounts of TNF-α secreted during pregnancy can stimulate apoptosis in human amniotic cells and syncytiotrophoblasts, leading to thrombosis and inflammatory response, blood vessel injury, and embryonic death [28]. Although without significant difference, the downtrend observed also showed that TNF-α might play a regulatory role in many aspects of the reproductive process.

A study suggested that IFN-γ produced by NK cells during mouse pregnancy is an important factor in the maintenance of normal pregnancy [29]. The present study showed that in late pregnancy, IFN-γ levels in serum were increased gradually as the number of pregnancy days increased. In addition, IFN-γ also enhances apoptosis in amniotic cells and trophoblasts activated by TNF-α, induces the activity of lymphokine-activated killer cells(LAK cells) treated with IL - 2, and promotes interleukin-2 receptor(IL- 2R) expression in T cells, which play an important role in the immune response [30, 31]. It is speculated that the mechanism of action might be a dynamic equilibrium process of maternal and fetal immune response and regulation from pregnancy to delivery. The gradual increase in the level of IFN-γ, as a Thl-type cell factor, plays an immune protection role at delivery.

During normal pregnancy, Th1/Th2 has a Th2 pattern shifted to humoral immunity, the so-called “Th2 phenomenon”, which means that maternal body during pregnancy tends to develop humoral immunity with the involvement of Th2-type cytokines, avoiding cellular immunity with the involvement of Th1-type cytokines [32]. When this ratio shifts in favor of Th1-type cytokines, damage of placental trophoblast cells and fetus might occur, leading to abortion [33]. Recent studies have found that in a variety of pathological pregnancies, such as pregnancy-induced hypertension syndrome and recurrent spontaneous abortion (RSA), the Th1/Th2 pattern in peripheral blood and placenta of patients is shifted to Th1 [34, 35]. Successful pregnancy is associated with increased levels of Th2-type cytokines secreted by lymphocytes [36–38]. This indicates that local immunity at the mother-fetal interface and the Th1/Th2 balance in maternal immune system play important roles in maintaining immune balance during normal pregnancy. IFN-γ, TNF-α and IL-6 belong to Th1-type cytokines [39]. By analyzing changes in serum IFN-γ, TNF-α and IL-6 amounts in pregnant rats, this study found that as the number of pregnancy days increased, IFN-γ, TNF-α and IL-6 amounts were significantly decreased in comparison with control levels, which fully demonstrates that the process of pregnancy is indeed dominated by Th2-type cytokines.

A series of physiological changes occurred in NK cells during pregnancy, which play an immunomodulatory role in endometrium by secreting a variety of cytokines, and participate in embryo implantation, growth and development, as well as early placental formation. A study found that the number of NK cells increases from the 1st week of pregnancy, peaking in the 3rd month of pregnancy, and gradually decreases, indicating that maternal cellular immune function during pregnancy is in a certain degree of inhibitory state [40]. In addition, Matsubayashi et al. found that the killing activity of NK cells in maternal peripheral blood is negatively correlated with pregnancy outcome [41]; the stronger the killing activity of NK cells in maternal peripheral blood before pregnancy, the higher the abortion odds, indicating that appropriate inhibition of the killing activity of NK cells in maternal peripheral blood during pregnancy is beneficial to the maintenance of pregnancy, as shown in this study that the total activity of NK cells increases in early pregnancy, and decreases in mid- and late pregnancy [42, 43].

In a word, changes in the concentrations of 23 cytokines and 7 hormones in serum samples from pregnant rats at the 14 time points were detected in this study, and variation trends of levels of all cytokines and hormones before and during pregnancy, as well as antenatal and postpartum were obtained. A directed and weighted network was used to quantitatively evaluate changes in intercellular wireless communication network between mother and fetus. This network could clearly reveal the process and pattern of wireless communication between mother and fetus through the placenta. In addition, by introducing parameters such as the output strength (*S*_*out*_), input strength (*S*_*in*_) and total strength (*S*_*total*_) of nodes, as well as overall network strength (S_*net*_), the wireless communication between mother and fetus during the whole pregnancy could be quantitatively described. Monitoring, diagnosis and treatment of fetal growth, development, differentiation and health based on changes in characteristic signaling molecules in maternal body at various time points could provide a new method and tool for maintaining maternal and embryonic health during pregnancy, ensuring normal delivery.

## Conclusions

In early pregnancy, two important immune transformations occur. The first occurs at day 5, i.e. Th1 transformation into Th2. In order to prevent fetal abortion, maternal innate and cellular immunity are inhibited, and embryo implantation as well as subsequent development and differentiation are protected; therefore, humoral immunity is enhanced significantly, which promotes the secretion of IgG, which crosses placental barrier through FcγRn and is transported to fetus, providing fetal protection. At day 7, the fetal heart is fully developed, and fetus has circulatory system which provides a platform for cellular signal communication. At this time, the second transformation occurs: the maternal immune system provides a signaling molecule for the embryonic circulatory system to promote Th2 transformation into Th1 as well as the development and differentiation of fetal innate and cellular immune cells, playing roles in immune recognition, response, and protection of embryonic innate immune cells mediated by maternal IgG (ADCC).

Characteristic signaling molecules throughout whole pregnancy were MCP-1, IL-10 and IL-13 (day 5), IL-1ɑ and IP-10 (day 7), RANTES (day 17), TSH (day 19), IL-2, IL-6, IL-12p70 and IL-18 (day 21). These findings indicated that diagnosis, prevention and treatment of fetal growth, development, differentiation and health could be monitored by detecting changes in concentrations of specific signaling molecules in maternal serum at various time points. However, further investigation is required to explore whether these findings are applicable to humans.
